# Supplementary material for: Conditional chemoconnectomics (cCCTomics) as a strategy for efficient and conditional targeting of chemical transmission
Source: eLife. 2024 Apr 30;12:RP91927. doi: 10.7554/eLife.91927 (PMC11060718; doi:10.7554/eLife.91927)
Supplement: Figure 3—source data 3. [file elife-91927-fig3-data3.zip › Figure 3 source data3 part 1.zip!/Figure 3-Source data 3-part1/1071f2-1-CRISPResso_Report_ztwktvfp.zip!/CRISPResso_on_1071f2_3/1c.Alignment_barplot.pdf]

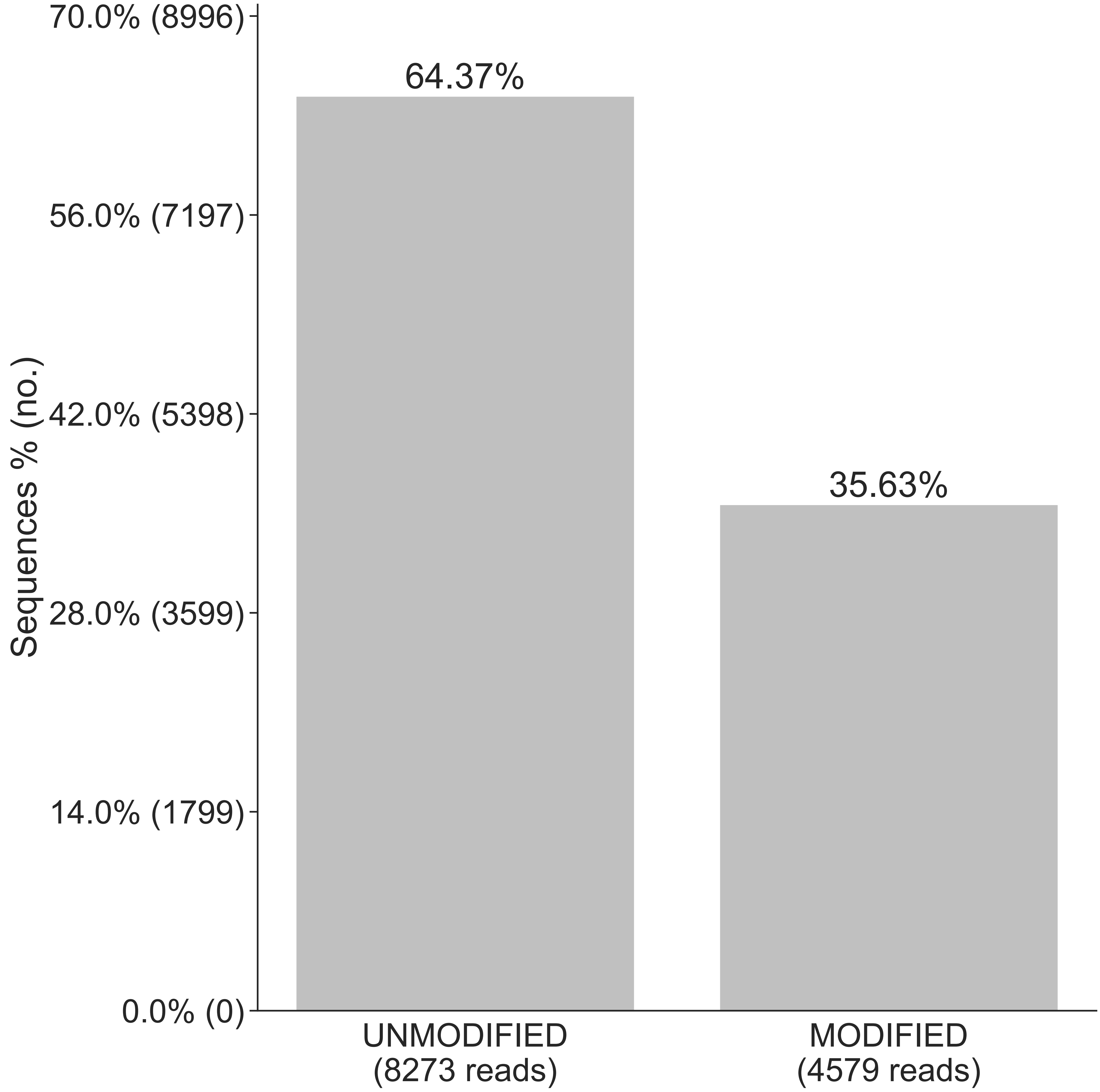

Sequences % (no.)
70.0% (8996)
56.0% (7197)
42.0% (5398)
28.0% (3599)
14.0% (1799)
0.0% (0)
64.37%
35.63%
UNMODIFIED
(8273 reads)
MODIFIED
(4579 reads)
